# 增强免疫组化和原位杂交方法检测非小细胞肺癌的ALK重排的临床可行性

**DOI:** 10.3779/j.issn.1009-3419.2015.02.04

**Published:** 2015-02-20

**Authors:** 辉 孟, 献争 高, 岚 张, 芳 刘, 文才 李

**Affiliations:** 1 450052 郑州，郑州大学第一附属医院病理科 Department of Pathology, The First Affiliated Hospital of Zhengzhou University, Zhengzhou 450052, China; 2 610041 成都，辉瑞肿瘤医学事务部 Pfizer Oncology Medical Affairs, Chengdu 610041, China

**Keywords:** 肺肿瘤, 间变性淋巴瘤激酶, 增强免疫组化, 荧光原位杂交, Lung neoplasms, ALK, Ventana-IHC, FISH

## Abstract

**背景与目的:**

继表皮生长因子（epidermal growth factor receptor, *EGFR*）突变之后，间变性淋巴瘤激酶（anaplastic lymphoma kinase, *ALK*）基因重排的非小细胞肺癌（non-small cell lung cancer, NSCLC）已经成为了肺癌的又一重要的临床分型。在临床上需要选择一种特异，灵敏并且价廉的方法 < 准确快速地找到ALK阳性的NSCLC患者。为此本研究探讨增强免疫组化法（ventana-IHC, V-IHC）检测ALK重排的临床可行性。

**方法:**

利用V-IHC检测172例NSCLC患者*ALK*重排，阳性患者以荧光原位杂交法（fluorescence *in situ* hybridization, FISH）验证。

**结果:**

172例NSCLC患者中有12例为ALK阳性，经过FISH验证，11例患者为阳性，符合率为91.7%。

**结论:**

在NSCLC中，V-IHC是ALK检测切实可行的方法，适用于*ALK*重排的NSCLC的筛查和诊断。

## Abstract (en)

**Background and objective:**

Besides epidermal growth factor receptor (*EGFR*) mutation, the non-small cell lung cancer (NSCLC) of anaplastic lymphoma kinase (*ALK*) rearrangement becomes another important clinical subtype. A specific and high-sensitive and economical detection way is convenience for identification of ALK positive NSCLC quickly and accurately. So the objective of our research is to detect *ALK* rearrangement in 172 cases of NSCLC by using enhancing immunohistochemical way (ventana-IHC, V-IHC).

**Methods:**

*ALK* rearrangement in 172 NSCLC samples was detected by using V-IHC, and positive staining cases were further verified by fluorescence *in situ* hybridization (FISH).

**Results:**

Among 172 NSCLC cases, there were 12 positive staining. The positive results were confirmed by FISH and 11 cases were FISH positive. The overall concordance between V-IHC and FISH is 91.7% (11/12).

**Conclusion:**

The V-IHC method is a reliable method for ALK arrangement and could be used in clinical screen and diagnosis.

间变性淋巴瘤激酶（anaplastic lymphoma kinase, ALK）是一种酪氨酸蛋白激酶，最早在间变性淋巴瘤的一个亚型中被发现，故得此名。2007年发现在非小细胞肺癌（non-small cell lung cancer, NSCLC）中，由于染色体易位引起间变性淋巴瘤激酶（anaplastic lymphoma kinase, *ALK*）重排，导致棘皮动物微管相关蛋白4（echinodermmicro tubule associated protein like 4, *EML4*）-*ALK*融合基因形成，催化ALK激酶持续活化，激活下游信号通路，刺激肿瘤细胞的无限制增殖^[1]^。继表皮生长因子受体（epidermal growth factor receptor, *EGFR*）突变之后，*ALK*重排是又一个NSCLC的驱动基因，*ALK*重排NSCLC已经成为了肺癌的一种新的亚型。这类患者约占5%-7%的NSCLC^[2]^，通常预后较差^[3]^。临床上*ALK*重排的检测方法主要有荧光原位杂交（fluorescence *in situ* hybridization, FISH）、免疫组织化学（immunohistochemistry, IHC）和逆转录-聚合酶链扩增法（reverse transcription-polymerase chain reaction, RT-PCR）^[4]^。其中FISH和RT-PCR是在基因层面上检测*ALK*重排，IHC是在蛋白表达层面上检测ALK重排。各种方法的灵敏度、特异性以及对标本的需求等各有不同^[4, 5]^。FISH和PCR的灵敏度和特异性较高，适用于ALK诊断，但是这两种方法的费用较高，检测时间长。RT-PCR还需要新鲜组织，对标本需要量大。常规IHC费用较低，检测时间短，但是结果的判读有一定的主观性，可用于ALK阳性NSCLC的筛查，阳性结果需要FISH或PCR验证。增强免疫组化法（enhancing ventana-IHC, V-IHC）采用了特异性很强的抗ALK（D5F3）的兔单克隆抗体结合增强DAB染色液和增强扩增试剂盒的检测体系，使用BenchMark XT平台进行自动化染色，洗片，背景干净，阳性信号强，结果容易判定。为了在临床上准确快速地找到ALK阳性的NSCLC患者，选择一种特异，灵敏并且价廉的方法非常重要。为此本研究将利用V-IHC检测本中心172例NSCLC患者的*ALK*重排，阳性的患者进一步使用FISH进行验证，比较V-IHC和FISH检测方法的相关性，分析V-IHC作为临床NSCLC患者*ALK*重排常规诊断方法的可行性。

## 1 资料与方法

### 1.1 临床样本采集

收集郑州大学第一附属医院病理科2014年1月-2014年5月172例NSCLC患者组织标本，所有标本来源于经皮肺穿刺术、纤维支气管镜和（或）颈部、锁骨上淋巴结活检和手术标本，且病理诊断为NSCLC。

### 1.2 样本处理流程标本处理

标本经10%中性福尔马林固定6 h-24 h，常规标准自动脱水，石蜡包埋。切片厚约4 µm，用于HE染色、V-IHC和FISH检测。V-IHC检测使用正电荷载玻片。

### 1.3 HE染色

采用HE染色观察组织细胞形态，细胞核染色呈深蓝色，胞浆染色呈深浅不同的粉红色，显示各种组织细胞成分的形态结构特点。

### 1.4 V-IHC检测

参照V-IHC试剂盒说明书进行。采用抗ALK（D5F3）兔单克隆抗体试剂（免疫组织化学法）染色对福尔马林固定、石蜡包埋的NSCLC组织切片进行临床评估，使用增强DAB染色液和增强扩增试剂盒，在VENTANA BenchMark XT平台进行染色。分别选取组织切片用于抗ALK（D5F3）兔单克隆抗体试剂（免疫组织化学法），兔单克隆阴性质控抗体，ALK阳性NSCLC病例及ALK阴性NSCLC病例作为系统水平质控，以确保染色仪器系统和相关检测试剂的性能合乎要求。如果这种系统水平质控不合格，就应重新染色。

### 1.5 FISH法检测*ALK*融合基因

参照原位杂交试剂盒说明书（vysis ALK-FISH-break-apart Kit）进行，略加改进。使用前，先于病例组与对照组的HE染色标本中找到有癌灶部位。在找到的癌灶部位利用靶基因DNA探头对标志基因进行检测。具体步骤：切片预处理：4 μm-5 μm石蜡切片65 ℃烤片3 min，常规脱蜡至水，滴加已复温的酶预处理液，室温孵育5 min-10 min，蒸馏水洗3次，每次2 min，中性甲醛室温固定10 min。梯度乙醇脱水（75%、85%、100%乙醇各2 min脱水），自然干燥或电吹风吹30 s。变性与杂交：每片滴加10 μL ALK探针混合物，立即用专用杂交盖片覆盖81 ℃变性6 min-10 min，42 ℃，杂交过夜（超过10 h）; 洗涤：浸入0.4×SSC/0.3%NP-40洗涤液1.5 min，后浸入2×SSC/0.1%NP-40洗涤液0.5 min。70%乙醇洗涤5 min，自然干燥。10 µL-15 µL DAPI复染。

### 1.6 结果判读

根据V-IHC试剂盒说明书，ALK阳性肿瘤细胞的特点是任何百分比的肿瘤细胞的胞浆内有很强的颗粒状染色。FISH法参照原位杂交试剂盒说明书，*ALK*重排的肿瘤细胞特征是橘红色和绿色信号互相分离，间距至少超过2个信号直径。无*ALK*重排的肿瘤细胞特征是橘红色和绿色信号重合为黄色或者相互粘合。FISH阳性结果的判定标准是计数50个肿瘤细胞，如果至少有25个（50%）的细胞存在信号分离，直接判定为阳性。如果信号分离的细胞仅为10%-50%，则需要重复计数50个肿瘤细胞，累加100个细胞中至少15个细胞存在信号分离（≥15%），则为ALK阳性。

## 2 结果

### 2.1 NSCLC

*ALK*重排的V-IHC检测采用系统水平质控排除非特异性背景染色。这些非特异染色包括：肺泡巨噬细胞中浅色的胞浆点状着色，神经来源的细胞（神经细胞和神经节细胞）和腺上皮染色，淋巴细胞浸润中一些散在的淋巴网状细胞及NSCLC中正常粘液（包括粘蛋白）内以及坏死区域的染色。ALK阳性的判定标准是任何百分比的肿瘤细胞内很强的颗粒状胞浆染色，在整个肿瘤中有着一致的强度（图 1）。利用以上标准判定检测在172例NSCLC患者有12例为*ALK*重排阳性，阳性率为6.98%。阳性标本中男性8例，女性4例。组织学分型8例为腺癌，4例为鳞状细胞癌（图 2），其中有1例为鳞状上皮原位癌。在这些ALK阳性病例中，肿瘤临床分期66.7%（8/12）为肺癌中晚期（Ⅲ期-Ⅳ期）。*ALK*融合基因阳性NSCLC患者的临床病理特征见表 1。

**1:**
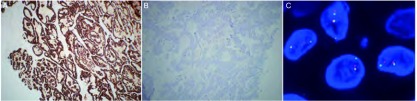
肺腺癌ALK增强免疫组化染色及FISH染色。肺腺癌组织增强免疫组化染色显示ALK蛋白表达阳性（A：阳性，×100），ALK阳性肿瘤病例中存在很强的颗粒状胞浆染色，信号均匀分布，在整个肿瘤部分中有着一致的强度；兔单克隆阴性质控抗体对照图片（B：×100），A、B图显示为同一个肿瘤组织标本；FISH检测（C：阳性，×1, 000）。 *ALK* fusion gene in lung adenocarcinoma (V-IHC and FISH). *ALK* fusion protein positive in lung adenocarcinoma (A: positive, ×100), strong granular cytoplasmic staining exists strong tumor cases, with the uniform signal distribution and the same intensity in the whole tumor section; Rabbit monoclonal antibody negative quality control section (B: ×100). A, B display tissue specimens for the same tumor; FISH detection (C: positive, ×1, 000).

**2:**
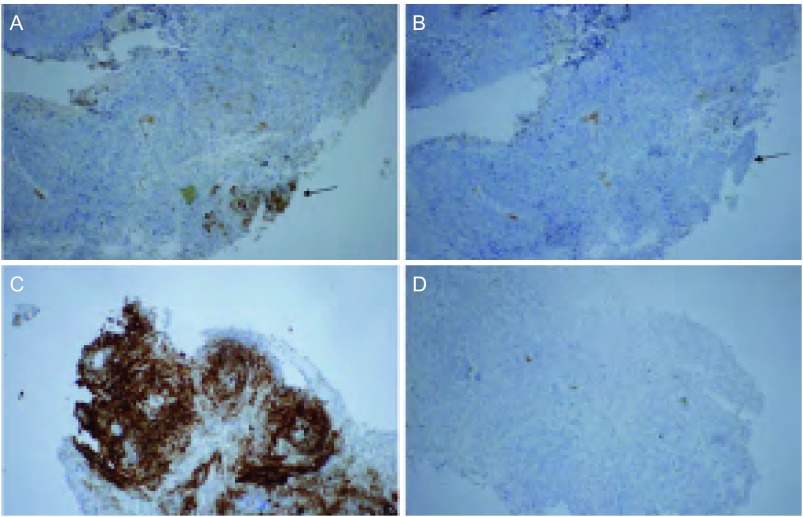
肺鳞癌*ALK*融合基因增强免疫组化染色（×100）。A：肺鳞癌增强免疫组化染色ALK阳性癌灶部位（箭头所示）；B：图片A的单克隆阴性对照图片（箭头所示为同一癌灶部位）；C：肺鳞癌增强免疫组化染色强的颗粒状着色，阳性效果显著；D：图片C的单克隆阴性对照图片。 *ALK* fusion gene in lung squamous cell carcinoma using V-IHC staining way (×100). A display ALK positive of V-IHC staining in pulmonary squamous cell carcinoma (arrow); B shows a monoclonal negative control section of A (arrow shows the same position of the squamous cell carcinoma tissue); C: Strong V-IHC positive granular staining of ALK in lung squamous cell carcinoma; D: Rabbit monoclonal antibody negative quality control section of C.

**1:** 172例非小细胞肺癌患者的临床和病理特征 Clinical and pathological characteristics of 172 cases of non-small cell lung cancer

Clinical and pathological characteristics	Number	ALK fusion protein positive (%)	ALK fusion protein negative (%)
Gender			
Male	93	8 (8.6)	85 (91.4)
Female	79	4 (5.1)	75 (94.9)
Age (yr)			
≥60	98	8 (8.2)	90 (91.8)
< 60	74	4 (5.4)	70 (94.6)
Smoking			
Yes	78	7 (9.0)	71 (91.0)
No	94	5 (5.3)	89 (94.7)
TNM stage			
Ⅰ-Ⅱ	76	4 (5.3)	72 (94.7)
Ⅲ-Ⅳ	96	8 (8.3)	88 (91.7)
Histology			
Carcinoma seraomatodes	6	0 (0)	6 (100.0)
Adenocarcinoma	100	8 (8.0)	92 (92.0)
Squamous carcinoma	58	4 (6.9)	54 (93.1)
Metastatic tumor from other place	8	0 (0)	8 (100.0)
Specimen type			
Biopsy	154	9 (5.8)	145 (94.2)
Excision of tissue	18	3 (16.7)	15 (83.3)

### 2.2 FISH验证

对V-IHC检测到的12例ALK阳性的患者标本行FISH验证，阳性判定的标准是计数100个肿瘤细胞，至少有15个细胞呈现红绿信号的分离，且距离超过2个信号直径（图 1C）。根据标准判定11例患者为FISH阳性，1例为FISH阴性，符合率为91.7%（11/12）。这例FISH阴性的患者（鳞状细胞癌）V-IHC染色呈现出染色不均的特点（图 2A）。

## 3 讨论

在*ALK*重排被发现为NSCLC的驱动因素的4年后，ALK激酶抑制剂-克唑替尼（Crizotinib，中文商品名：赛可瑞）于2011年在美国被批准治疗ALK阳性的局部晚期和转移性NSCLC。克唑替尼一线治疗的客观缓解率（objective response rate, ORR）为74%，中位无疾病进展生存期（median progression free survival, m-PFS）为10.9 mo。二线治疗的有效率为50%-60%，m-PFS为7.0 mo^[6-8]^。随着靶向药物克唑替尼2013年在中国上市，面对中国每年肺癌的新发病例接近35, 000例的现状，临床上迫切需要寻找经济简便的方法，准确快速地找到ALK阳性的NSCLC患者。由罗氏公司开发的V-IHC诊断试剂盒已经在欧洲和中国获批用于诊断ALK阳性NSCLC。由于其自动化操作，可使检测流程和判读得以标准化。该技术平台使用了基于非内源性半抗原，信号扩增多聚体和辣根过氧化酶（HRP）的染色信号方法技术，在不影响特异性的前提下，提高了ALK检测的灵敏度。结果判读采用二分类，即仅为阴性和阳性，阳性结果即可诊断为ALK阳性NSCLC。有研究证实V-IHC与FISH结果的吻合率为98.8%，判读的重复性为99.7%。本中心利用V-IHC方法对172例NSCLC患者行ALK检测，有12例为ALK阳性。阳性的标本再经过FISH验证，11例为FISH阳性，V-IHC和FISH检测*ALK*重排的符合率91.7%。而且将12例阳性病例进行EGFR突变检测，结果均为野生型，数据未提供。

对于V-IHC检测ALK阴性的标本是否需要FISH验证？有研究^[9]^比较了217例经过V-IHC检测为ALK阴性的患者行FISH验证，同样也是ALK阴性。基于该研究数据和出于检测费用的考虑，本研究对V-IHC检测ALK阴性的标本没有再行FISH验证。

与常规IHC手动洗片不同，V-IHC采用强度较大的机器洗片，以减少背景干扰。为了避免标本在洗涤过程中脱落，推荐使用正电荷载玻片，切片厚度为3 µm-5 µm。在V-IHC检测为ALK阳性的标本中有1例存在着染色不均的现象（图 2A、图 2B）。本例FISH检测时可以计数到足够数量的肿瘤细胞，但是结果为FISH阴性。推测可能存在荧光原位杂交探针未覆盖或染色过程中脱片等原因，也可能和肿瘤异质性有关。

为了减少染色不均的现象，固定液必须是10%中性福尔马林，在操作上确保固定时间不要低于6 h，组织块与固定液要充分接触。建议外科医生在固定之前对手术大标本切割2刀-3刀，使标本充分浸泡于固定液中。肿瘤异质性指的是肿瘤不同部位，或者原发瘤和转移瘤的基因表达不一致的现象。为了避免肿瘤异质性对检测结果的影响，最好对肿瘤多点取样同步检测。V-IHC可在亮视野中观察，对于观察染色不均及肿瘤异质性的标本V-IHC较FISH有一定优势，如图 2。

对于V-IHC染色不均的患者，在标本足够的情况下最好采用FISH验证。但是FISH的判读必须要计数100个肿瘤细胞，至少有15%的阳性细胞才能判定为阳性。有时候小活检标本量太少，难以满足100个细胞的计数，可能会影响FISH的判读，导致判断为FISH阴性。

*ALK*重排在东亚地区肺癌患者中的发生率约为3%-7%^[10]^。本研究发现*ALK*重排在中国患者中为6.89%，接近于东亚人群。ALK阳性的NSCLC以腺癌为主，鳞癌非常少见^[11]^。我们研究发现6.9%的鳞癌患者为ALK阳性，高于文献的报道。推测可能为鳞癌组织里混有腺癌的成分。进一步重复检测，排除了腺鳞癌的可能。由于该研究样本量比较小，难以反映鳞癌中ALK阳性比例，需要加大样本量予以验证。无论怎样，ALK阳性的鳞癌患者也是克唑替尼的适用人群。

总的来说，V-IHC灵敏度和特异性高于常规IHC，结果容易判读。V-IHC与FISH比较吻合度高，价格便宜，检测时间短。标本用量小，仅需要1-2张石蜡切片。V-IHC是NSCLC的ALK检测切实可行的方法，适用于ALK阳性NSCLC的诊断。
